# Upper limb compartment syndrome requiring fasciotomies following a *Vipera ammodytes* (horned viper) snakebite: a case report

**DOI:** 10.1080/23320885.2026.2662057

**Published:** 2026-04-25

**Authors:** Bisera Nikolovska, Elizabeta Miovska, Jasmina Dobrevska, Emilija Markovikj, Marko Spasov

**Affiliations:** ^a^ Ss Cyril and Methodius University in Skopje, University clinic for plastic and reconstructive surgery, Skopje, Republic of North Macedonia; ^b^ Ss Cyril and Methodius University in Skopje, Faculty of Medicine, Skopje, Republic of North Macedonia; ^c^ Ss Cyril and Methodius University in Skopje, University Clinic for Traumatology, Orthopedic Diseases, Anesthesia, Resuscitation, Intensive Care, and Emergency Center, Skopje, Republic of North Macedonia

**Keywords:** Snakebite, horned viper, *Vipera ammodytes*, compartment syndrome, fasciotomies

## Abstract

The clinical severity of snakebite envenomation depends on several factors, including the snake species, the amount of venom injected, the site of envenomation, the timing of antivenom administration, and patient-specific factors. Compartment syndrome is a rare but serious complication of severe envenomation, caused by toxin-induced tissue damage and swelling. We present a case of a 71-year-old man with extensive swelling and ecchymosis of the right upper limb, signs of hand ischemia, and systemic effects, including hypotension, anuria, bradycardia, bradypnea, and somnolence, two days after a Vipera ammodytes (horned viper) bite to his right forearm. Delayed initial treatment and antivenom administration (six hours post-bite due to the heavy influence of alcohol) aggravated the clinical course. Emergency fasciotomies were performed to decompress all compartments of the upper limb. Wound closure was achieved with progressive tension closure, split-thickness skin graft, and platelet-rich plasma (PRP) to the volar side of the forearm. The patient achieved complete recovery with full functional restoration of the upper limb. Post-snakebite compartment syndrome (PSCS) results from a multifactorial cascade of venom-induced local tissue injury, leading to increased intracompartmental pressure and microvascular ischemia. Prompt surgical treatment is necessary if symptoms persist or worsen despite antivenom administration.

## Highlights

- Snakebites from *Vipera ammodytes* may cause severe envenomation and may result in serious complications and death.
- Compartment syndrome is a rare but serious complication of severe envenomation, caused by toxin-induced tissue damage and swelling.
- Surgical decompression may be necessary to preserve limb function and prevent long-term complications if symptoms persist or worsen despite antivenom administration.

## Introduction

Snakebites are a global yet often overlooked public health issue that the WHO has recognized as a neglected tropical disease since 2017. An estimated 5.4 million bites occur each year, leading to 2.7 million envenomations and between 81,000 and 138,000 deaths worldwide. Complications include tissue necrosis, paralysis, bleeding, and kidney failure, with 400,000 cases of permanent disability or amputations reported [1].

In North Macedonia, there are 16 different snake species, three of which are venomous and pose a threat to humans: the horned viper (*Vipera ammodytes*), the common European adder (*Vipera berus*), and the meadow viper (*Vipera ursinii*). Of these, the horned viper is the most significant in the region [2]. No publicly available national registry or centralized database specifically tracks snakebite incidence and severity in North Macedonia. Data remains limited to sporadic case reports, veterinary studies, and regional European overviews; no official government statistics have been identified [1]. A study from Western Serbia, a neighboring region with similar fauna, reported 249 cases over 13 years, approximately 19 per year, with no fatalities [3]. The hand was the most common bite site (52%), followed by the foot (38%) [4]. Data from a similar study conducted in Croatia showed that 632 people were hospitalized due to venomous snakebites over a 21-year period (1998–2019), with 3 deaths reported (mortality rate 0.2% per year). The authors emphasize that these numbers are underreported due to limited healthcare access or reliance on traditional medicine among snakebite victims [5]. The yearly incidence of *Vipera ammodytes* bites in Central and Southeastern Europe is 1.61 per million people, which is higher than that for the common European adder (1.00 per million). *Vipera ammodytes* bite results in higher grade severity and more antivenom needed (72%) versus *Vipera berus* bites(39%) [6].

Snakebite (ophidism) is a condition caused by the injection of toxic substances secreted by snake glands through a bite. Most snakebites occur accidentally, often during agricultural or recreational activities, and are more common during the summer months. The upper extremity is the most common bite site (53,1%). Venoms of *Vipera* species contain diverse proteins and enzymes, including metalloproteinases, phospholipases A2, serine proteases, and C-type lectins. These venoms are mainly hemotoxic and cytotoxic, with neurotoxic components in some species (*Vipera ammodytes* and *Vipera aspis)* [7].

The clinical severity of snakebite envenomation symptoms depends on multiple factors, including the snake species, the amount of venom injected, the site of envenomation, the timing of antivenom administration, and patient-specific factors. Symptoms can range from none to fatal; although rare, fatal outcomes can occur from neck bites, direct vascular inoculation, or extremity envenomation in elderly patients and young children, who are especially vulnerable to severe venom effects. Initial management includes: wound management, resuscitation, and antivenom administration ideally within 3 h, to neutralize circulating venom [8].

Compartment syndrome is a rare but serious complication following severe viper envenomation, caused by intense local swelling and venom-induced tissue damage. If not treated promptly, compartment syndrome can lead to muscle ischemia, necrosis, and permanent limb deformities such as Volkmann’s contracture. Early detection and prompt decompression are essential to preserve limb function [9].

We present a case of severe envenomation after a snake bite to the forearm, which resulted in compartment syndrome requiring urgent surgical intervention—fasciotomies. The patient had a delayed antivenom administration. This case report follows the SCARE checklist 2025 [4].

### Case presentation

**Figure 1.**
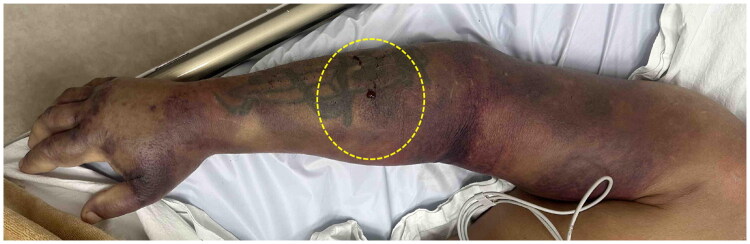
Yellow oval highlights the snake-bite site on the patient’s right forearm 48 h after injury. (Table 1).

A 71-year-old man was admitted to the University Clinic for Plastic and Reconstructive Surgery in Skopje two days after a *Vipera ammodytes* bite on the volar side of his right forearm while fishing (Figure 1). The snake was killed and identified as a horned viper. At the time of the incident, the patient was under the heavy influence of alcohol and therefore waited 6 h before seeking medical care at a local hospital (Public General Hospital - Gevgelija), with visible fang marks, severe pain, and swelling in his right forearm. He showed signs of envenomation, with manifestations difficult to distinguish from alcohol intoxication, as noted by the local doctor. Past medical history was significant for type 2 diabetes mellitus and alcohol use disorder. Laboratory results showed: WBC 29.4, CK 738, AST 63, LDH 266, and Gly 13.9. The patient received two vials of Antiviperinum Serum (VIEKVIN^®^, Torlak Institute, Serbia) intramuscularly, tetanus antitoxin, intravenous crystalloids, two antibiotics, NSAIDs, antihistamines, gastroprotective agents, and corticosteroids, and underwent initial wound management, including cleaning and dressing.

On day 2, he was transferred to our facility, a tertiary center, for surgical evaluation, further assessment, and treatment. (Table 1) Over the following 24 h, swelling progressed to involve the entire right forearm, arm, and hand, with increasing pain on both active and passive movements and cyanosis of the hand and fingers. Systemically, he became hypotensive, anuric, bradycardic, bradypneic, and increasingly drowsy despite symptomatic therapy. A second antivenom dose (two vials) was administered intravenously approximately 24–36 h after the bite, with no clinical improvement. He was therefore transferred to our tertiary center for further management.

**Figure 2.**
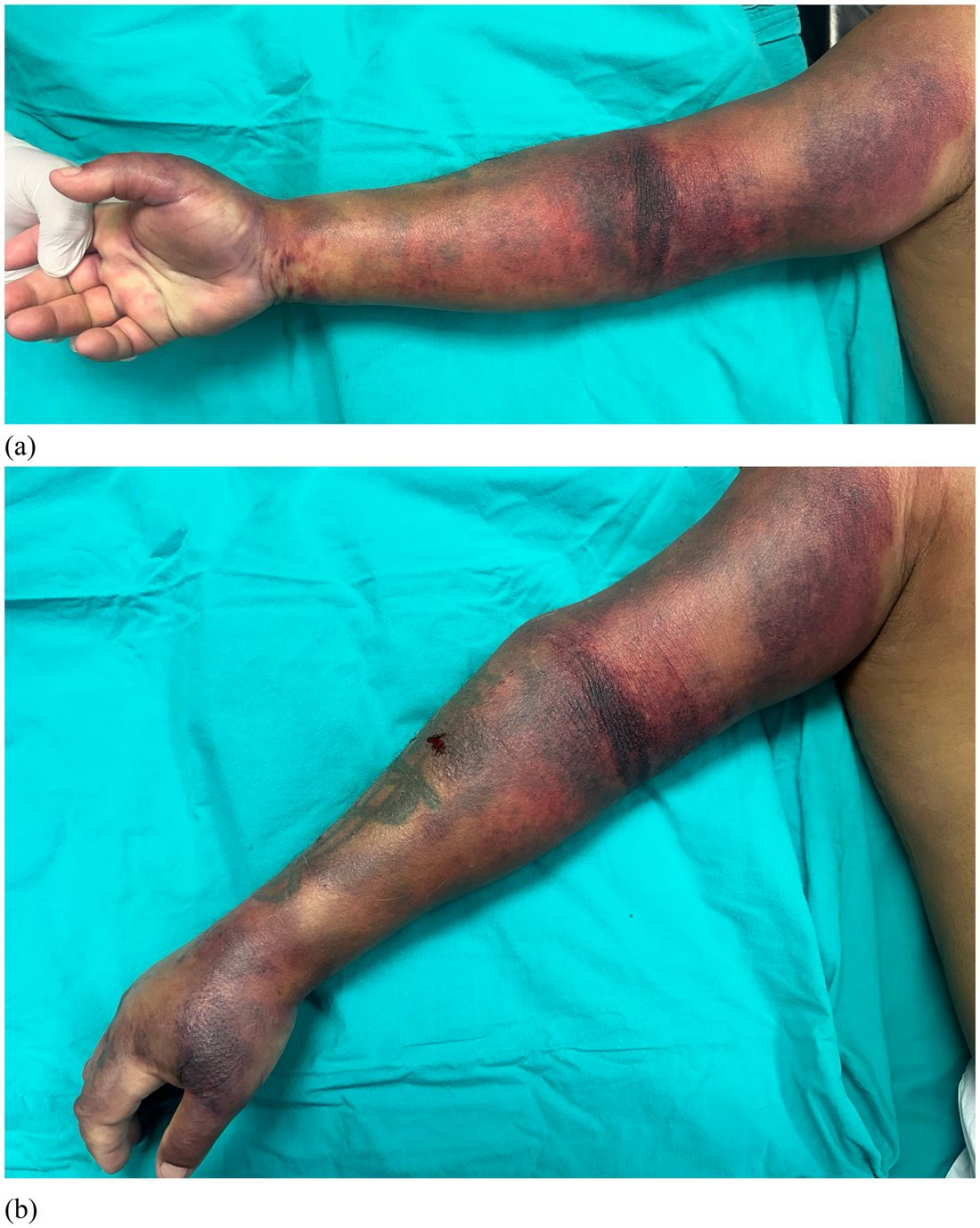
Preoperative photos: swelling and ecchymosis of the right upper limb. (a) volar side; (b) dorsal side.

On examination, the patient was afebrile (36.8 °C), drowsy, delusional, hypotensive (80/40 mmHg), anuric, bradycardic (42 bpm), and bradypneic (8 breaths/min). Bilateral mydriasis, ptosis, and facial edema were present. Examination of the right upper limb showed extreme swelling and ecchymosis; the fingers and palm were pale, and the right forearm and hand were extremely painful and firm on palpation. Active and passive movements of the elbow, wrist, and finger joints were severely limited by edema and pain. Sensory testing revealed decreased sensation over the ulnar and median nerve areas. The vascular exam showed diminished radial and ulnar pulses and delayed capillary refill (>3 s). A clinical diagnosis of compartment syndrome of the right upper limb was made (Figure 2).

The diagnostic workup included CT scans of the head, neck, and chest, which were unremarkable, and CT angiography of the upper limb, which showed diffuse soft-tissue edema and excluded pseudoaneurysm, active bleeding, hematoma, or venous thrombosis. Electrocardiography demonstrated sinus bradycardia with T‑wave flattening. Laboratory investigations confirmed hepatic and renal dysfunction, hyperfibrinogenemia, and coagulopathy (Table 2). Due to the lack of appropriate equipment, intracompartmental pressure was not measured.

**Table 1.** Timeline of patient management.

Timeline of patient management	
0 h	Snakebite on the right forearm, while fishing. The patient was intoxicated, and continued drinking according to his statement
6-12 h	Admited to a local hospital, presented with intoxication, severe forearm pain, swelling, and red-to-livide discoloration. Recieved two vials of Antiviperinum Serum (VIEKVIN^®^, Torlak Institute, Serbia) intramuscularly, tetanus antitoxin, intravenous crystalloids, two antibiotics, NSAIDs, antihistamines, gastroprotective agents, and corticosteroids and wound management
12-24 h	Local symptoms deteriorated progressively- the swelling increased and spread to the entire right forearm, arm, and hand; increasing pain exacerbated with active and passive movements, visible cyanosis of the hand and fingers, and facial oedema. Systemic symptoms also worsened, and the patient became hypotensive, anuric, bradycardic, bradypneic, and increasingly drowsy despite symptomatic therapy
24-36 h	two additional vials of Antiviperinum Serum (app.24-36h after the snakebite) intravenously
36-48 h	no improvement of symptoms was noted, transferred to a tertiary center for surgical evaluation and further treatment.
48-54 h	Examination, work-up, CT scans, medical treatment; follow-up serial exams of the upper limb hourly (for 3 h)
55 h	Emergency fasciotomies were performed to decompress all anatomical compartments of the upper limb.
Postoperative day 3	Primary closure for finger fasciotomy incisions. Placement of a vessel loop in a crisscross pattern over the wound edges of the volar fasciotomy (shoelace technique) for gradual closure.
Postoperative day 7	The patient underwent split-thickness skin grafting combined with platelet-rich plasma (PRP) to cover the volar fasciotomy wound of the forearm.
Postoperative day 20	Patient discharged from hospital; all wounds are healed.
Two months follow-up	The patient had achieved full functional recovery of the upper limb with an excellent scar appearance.

**Table 2.** Laboratory values during hospitalization.

Lab Test	Normal Range	Day 1	Day 2	Day 3	Day 4	Day 6	Day 8	Day 9	Day 10	Day 18
WBC	4.00-9.00 10^9/L	21.0	22.07	12.4	11.81	7.47	6.58	6.29	6.7	10.5
RBC	4.20-5.50 10^12/L	3.50	2.69	2.21	2.87	2.52	2.68	3.53	3.44	3.30
Hb	120–180 g/L	113	88	74	90	79	83	108	105	105
PLT	150-450 10^9/L	156	204	161	153	139	228	219	247	399
AST	10-34 U/L	44	37	37	41	45	56	46	35	17
ALT	10-45 U/L	21	20	20	21	21	32	30	26	12
Urea	2.7-7.8 mmol/L	17.8	20.6	23.3	21.5	13.3	7.4	7.8	5.1	1.7
Creatinine	45-109 µmol/L	419	463	400	283	125	93	84	74	76
PT	9.8-14.2 sec	12	14.3	13.5	11.8	11.1	11.7	12.1	12.5	13
aPTT	27.9-37.7 sec	26	25.4	34.1	28.4	27.1	25.1	25.6	26.1	25.4
D-Dimers	<0.5 µg/mL	1457	1698	1378	1449	1314	4328	4120	4101	5450
CRP	<6 mg/L	22.2	23.7	47.9	42.8	79.5	136.0	84.0	83.2	32.9
LDH	<248 U/L	289	304	276	289	242	319	342	269	189
CK	24–173 U/L	1243	1137	873	866	379	534	485	477	213
CK-MB	<25 U/L	60.71	53.29	38.46	31.70	22.12	25.93	25.30	25.32	24.28
Fibrinogen	2.3-3.5 g/L	6.1	5.8	5.4	4.3	3.7	3.2	/	/	/

*WBC, white blood cells; RBC, red blood cells; Hb, hemoglobin; PLT, platelets; AST, aspartate aminotransferase; ALT, alanine aminotransferase; PT, prothrombin time; aPTT, activated partial thromboplastin time; CRP, C-reactive protein; LDH, lactate dehydrogenase; CK, creatine kinase; CK-MB, creatine kinase MB*.

*“/” indicates test not performed as values had normalized and repeated testing was not clinically indicated*.

Medical treatment involved aggressive intravenous fluid resuscitation, vasopressor support (dopamine), corticosteroids, broad-spectrum antibiotics (ceftriaxone), osmotic diuretics (mannitol), analgesics, gastroprotective therapy, and antithrombotic prophylaxis. A third dose of antivenom was not administered, as recommended by the infectious disease specialist consulted on admission, who deemed it non-beneficial given the lack of response to the second dose and advocated supportive therapy, including inotropes.

Serial clinical examinations of the upper limb were performed hourly to assess pain, edema, neuro-vascular status, and skin color. After three hours of intensive medical treatment without improvement in limb status, urgent surgical decompression was indicated.

**Figure 3.**
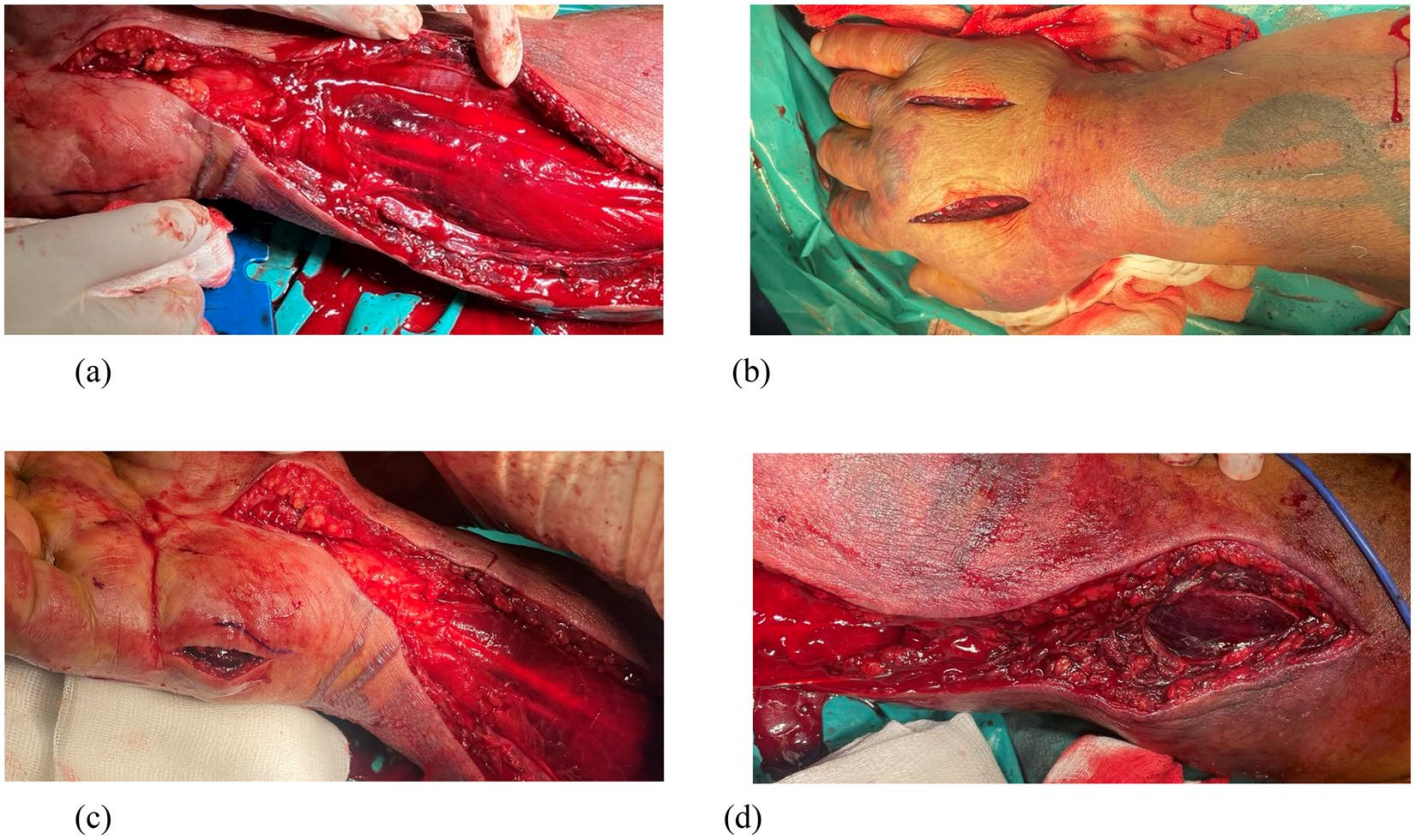
Edema and cyanotic discoloration of the muscles. (a)volar forearm fasciotomy; (b)fasciotomies of the dorsum of the hand; (c)fasciotomy of the hypothenar; (d) volar fasciotomy of the arm.

**Figure 4.**
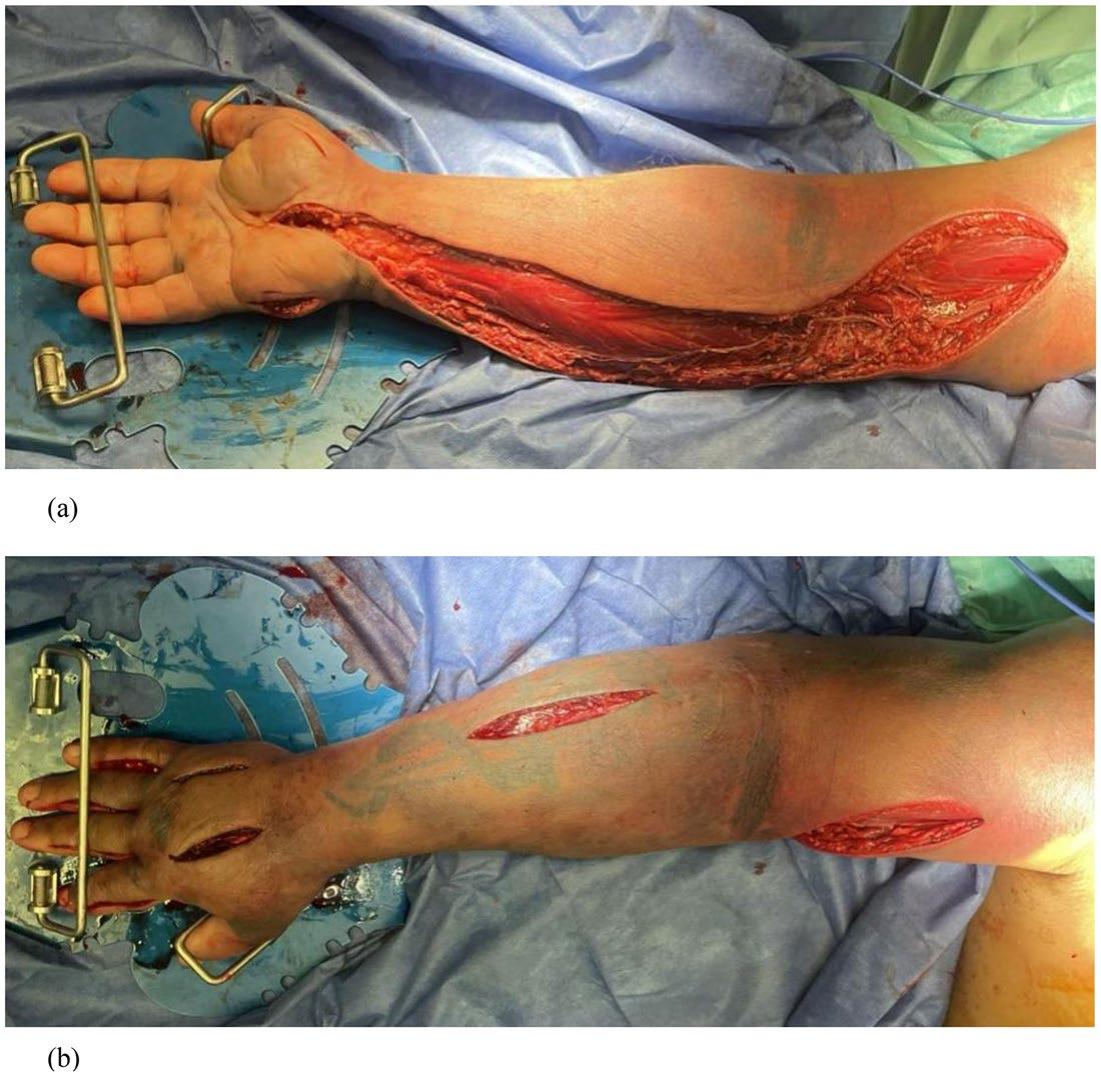
Intraoperative photos after completing the fasciotomies; skin and muscles have returned to their normal color. (a) Fasciotomy of the volar side of right upper limb; (b) faciotomy of the dorsal side of the right upper limb.

Under general anesthesia, emergency multi‑compartment fasciotomies were performed. Decompression included release of the carpal tunnel and Guyon canal, volar and dorsal compartments of the forearm and arm, intrinsic hand compartments, and all five digits *via* bilateral midlateral incisions. Intraoperatively, the muscles were markedly edematous and cyanotic (Figure 3). Within minutes of completing the fasciotomies, muscle color and consistency normalized, and palpable pulsations returned in the radial and ulnar arteries (Figure 4). The procedure and anesthesia were uneventful.

**Figure 5.**
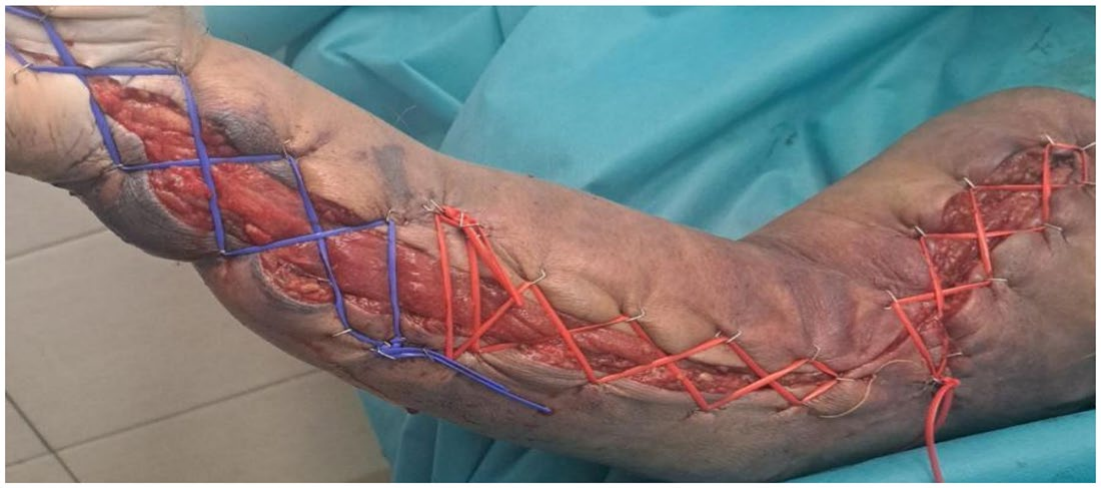
Progressive tension closure using the shoelace technique.

Postoperative care included intensive intravenous hydration, osmotic diuresis, and meticulous local wound management, with close monitoring of neurological, renal, hepatic, and respiratory function. A control brain CT on postoperative day 2 was requested because of persistent mydriasis and ptosis, and showed no abnormalities. The patient experienced episodes of hallucinations and agitation, attributed to a combination of envenomation and alcohol withdrawal, and was managed with antipsychotic medication. Primary closure was performed for the dorsal and finger fasciotomy incisions on postoperative day 3 after evident resolution of edema. The volar fasciotomy wound was gradually reduced in size by threading a vessel loop in a crisscross pattern over the wound edges and tightening it over time (shoelace technique). (Figure 5)

**Figure 6.**
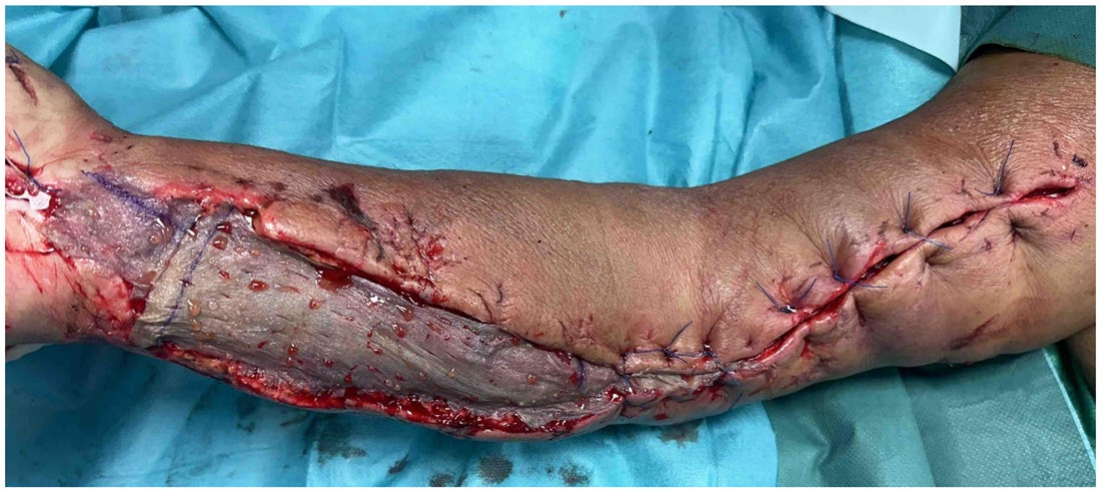
Split-thickness skin graft with PRP on the volar side of the forearm; Primary closure on the upper arm.

One week after the initial fasciotomy, the patient underwent split-thickness skin grafting combined with platelet-rich plasma (PRP) to cover the volar fasciotomy wound of the forearm. The skin graft was placed over the forearm defect, and freshly prepared platelet-rich plasma (ACP Arthrex) was applied to both the wound bed and the skin graft. The fasciotomy on the upper arm was suitable for primary closure as a result of the progressive tension closure. (Figure 6)

**Figure 7.**
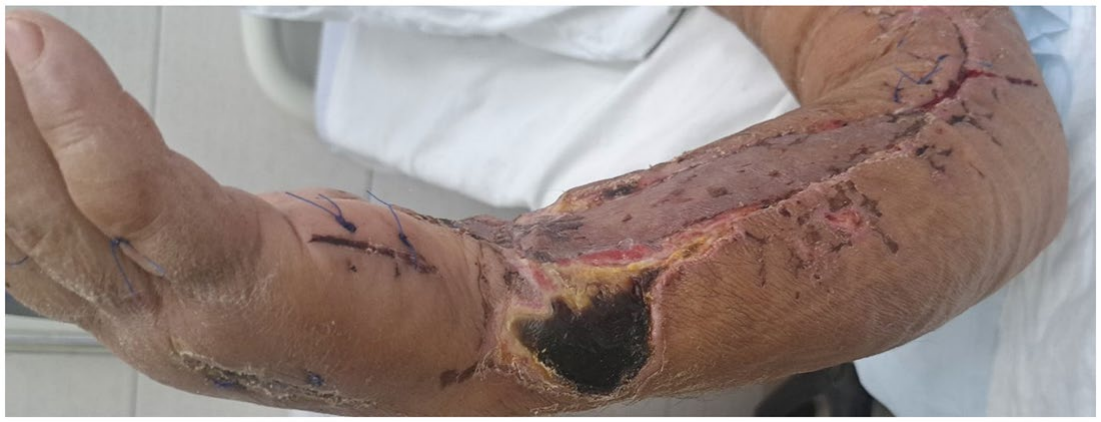
Skin necrosis on the distal part of the forearm.

**Figure 8.**
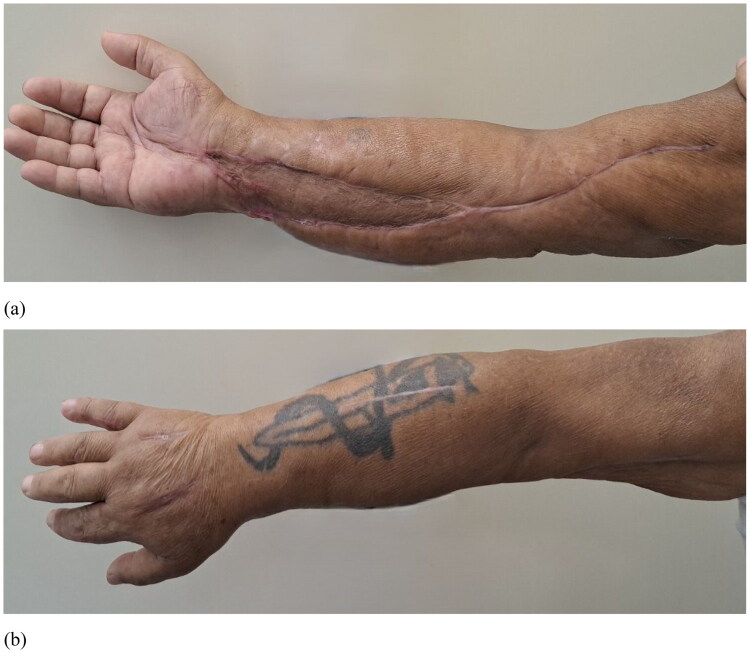
Two-month follow-up scar appearance: (a) volar side of the forearm and hand; (b) dorsal side of the forearm and handNote: the patient has a snake tattoo at the site of the snakebite/scar.

Postoperatively, hepatic and renal functions gradually improved. A small area of skin necrosis on the distal forearm was treated with advanced wound care and healed without additional surgeries (Figure 7). The patient underwent daily physical therapy. He was discharged from the hospital 20 days after admission. At first follow‑up, 7 days after discharge, he had a normal range of motion in the shoulder, elbow, wrist, and hand, good muscle strength and grip, and intact neurovascular status with satisfactory scars. At 2 months, he demonstrated full bilateral range of motion, normal sensation, Manual Muscle Testing of 5/5 in all muscle groups, and a grip strength of 39 kg (approximately 85% of the contralateral side), with an excellent cosmetic result (Figure 8).

## Discussion

*Vipera ammodytes* venom is dominated by metalloproteinases (SVMPs), phospholipases A2 (PLA2), snake venom serine proteases (SVSPs), and C-type lectins (CTLs), which together account for about 75% of the venom [10,11].

The clinical severity of envenomation depends on several factors, including the species involved, the amount of venom injected, the site of the bite, the timing of antivenom administration, and host comorbidities. It can lead to a wide range of signs and symptoms, from localized tissue damage to life-threatening systemic effects [8,9]. Our patient presented with symptoms classified as grade 3 ophydisam according to the Audebert-Boels classification [12,13]: altered mental status, hemodynamic instability, anuria, severe swelling and pain in the right upper extremity, with ecchymosis, paresthesias, and diminished radial pulse. Liver and kidney impairment were also evidenced by elevated urea, creatinine, CK, LDH, and AST. (Table 3)

**Table 3.** The Audebert-Boels classification system for snake-bite envenomation severity [13].

Score	Characteristics/Symptoms
0	No envenoming (“dry bite”): Fang marks only; no edema or local reaction
1	Minimal envenoming: Local edema around bite area; no systemic symptoms
2a	Moderate envenoming: Regional edema involving most of the limb, hematoma or adenopathy
2b	Grade 2a + moderate systemic symptoms (mild hypotension, vomiting, diarrhea, neurotoxic signs) and/or biological severity criteria (e.g. leukocytes >11,000/L, neutrophils >65%, INR >1.15)
3	Severe envenoming: Extensive edema spreading to the trunk; hemodynamic instability (shock, prolonged hypotension, bleeding)

Prompt initial care, including first aid and timely administration of antivenom, is essential in determining clinical outcomes. In optimal circumstances, antivenom should be administered as early as possible after snakebite, ideally within 3 h, to neutralize circulating venom and arrest the pathophysiological cascade [8,13]. Initial dosing depends on the severity of envenomation and may require repeated doses if symptoms persist or worsen [11,14]. In this case, the first antivenom dose was delayed- given 6 h after the bite, and a second dose 36 h later, by which time venom‑induced edema, capillary damage, and local hemorrhage had likely progressed beyond the point of reversibility. Furthermore, alcohol intoxication at the time of the snakebite and alcohol consumption afterwards are likely to worsen envenomation severity through vasodilation, accelerating venom spread, blunting early symptom recognition, and potentiating coagulopathy, contributing to the severe snake envenomation.

Post-snakebite compartment syndrome (PSCS) results from a multifactorial cascade of venom-induced local tissue injury, leading to increased intracompartmental pressure and microvascular ischemia. Upper extremity bites, especially to the hand and forearm, pose a higher risk of compartment syndrome than lower extremity bites due to the area’s rich blood supply, complex anatomy, and thinner skin and subcutaneous tissue, which increase the chance for venom to be injected directly into deep muscular compartments and to spread further rapidly [15].

Acute compartment syndrome(ACS) is primarily a clinical diagnosis based on the classic “5 Ps”: pain, pallor, paresthesia, paralysis, and pulselessness. Typical findings include pain out of proportion, pain with passive stretch, tense compartment, and progressive neurologic deficits (paresthesia, weakness). Vascular compromise with diminished pulse and skin color changes is a late finding. Intracompartmental pressure monitoring can provide objective confirmation, particularly in unconscious patients or those with equivocal examinations; pressures above 30–40 mmHg are commonly used thresholds for fasciotomy [16,17]. At our institution, pressure monitoring was unavailable, so the decision to perform fasciotomy relied entirely on serial clinical assessment. Given the coexistence of early and late signs of compartment syndrome, our threshold for surgical decompression was deliberately low. A CT angiography of the upper extremity was performed to exclude other acute vascular issues and showed non-specific signs such as tissue edema. However, CT angiography is not a standard diagnostic tool for acute compartment syndrome [18].

The study by Hsu et al. identified elevated white blood cell count and AST at presentation as independent predictors of PSCS in Taiwanese viper envenomation. Our patient’s marked leukocytosis and elevated AST are consistent with this pattern (Table 2), reflecting a strong inflammatory and tissue-injury response. However, these findings are derived from Asian viper species, and further work is needed to evaluate whether similar laboratory predictors apply to *Vipera ammodytes*-related PSCS.

The role of fasciotomy in PSCS remains controversial. Many authors recommend that fasciotomy be performed only after correction of coagulopathy, clear clinical evidence of compartment syndrome, and, when possible, documentation of elevated intracompartmental pressure. Reported fasciotomy rates vary widely between regions and species; a European review of *Vipera* snakebites reported fasciotomy in 4.2% of cases, whereas a Korean series of pit viper bites found fasciotomy necessary in 10.8% of patients when pressure monitoring was used routinely. The latter study suggests that systematic pressure measurement may reveal previously underdiagnosed cases of clinically significant compartment syndrome rather than reflecting inherent species differences [7,15].

Early fasciotomy, ideally within 4–6 h of symptom onset or clinical diagnosis, has been shown to reduce complications and facilitate functional recovery [19]. The mean interval from snakebite to fasciotomy in published case reports ranges from 1 to 48 h, with outcomes inversely related to delay [20–27]. We proceeded with early, extensive, complete multi‑compartment fasciotomy and this comprehensive approach achieved immediate reperfusion of the musculature and ultimately preserved full limb function. Prolonged compartmental upper limb ischemia inevitably leads to irreversible tissue necrosis and Volkmann’s ischemic contracture, yet this complication is entirely preventable through early diagnosis and prompt intervention [16,17]. One limb amputation has been reported despite fasciotomy, underscoring the severity of venom-induced tissue destruction [28].

Fasciotomy wounds pose a management challenge; studies report a 31% complication rate following fasciotomy, with long-term sequelae including dysesthesias, persistent swelling, and tethered scars observed in >75% of surgical patients [21]. Fasciotomy wounds after snakebite are even more challenging owing to the hostile wound environment created by venom-mediated proteolysis, tissue necrosis, and a high risk of bacterial contamination [7,8]. Studies demonstrate that the shoelace technique achieves faster wound closure (typically within 7–10 days), reduces hospitalization duration, and healthcare costs [29]. In our case, we managed to close the wound primarily on the upper arm and significantly reduce the size of the defect on the forearm before skin grafting.

Secondary infection is a common complication, with Staphylococcus aureus and Gram-negative organisms such as Escherichia coli frequently isolated, and infection rates reported between 10 and 32% [30,31]. We used PRP as an adjunct to split‑thickness skin grafting, based on evidence that PRP enhances angiogenesis and granulation, modulates inflammation, and exhibits antimicrobial properties in contaminated wounds [32–34]. This combined strategy likely contributed to uneventful healing and an excellent functional and aesthetic outcome.

In countries with lower socioeconomic development and limited medical resources, snakebite complication rates are significantly higher than in developed nations, underscoring the essential role of surgical expertise in managing envenomation-related complications [13,35].

## Conclusion

This case illustrates that *Vipera ammodytes* envenomation can rapidly progress to severe local and systemic toxicity, including post‑snakebite compartment syndrome, particularly when antivenom administration is delayed. Early recognition, repeated clinical assessment, and timely multi‑compartment fasciotomy are critical to preventing irreversible muscle and nerve injury and preserving limb function. These observations highlight the importance of maintaining a low threshold for operative intervention in patients with suspected post‑snakebite compartment syndrome when progressive swelling, neurovascular compromise, and ischemic signs, fail to improve after antivenom and supportive therapy, especially in resource‑limited settings where compartment monitoring is unavailable.

## Data Availability

All relevant data supporting the findings of this case report are included within the article. Additional information regarding this case may be available from the corresponding author upon reasonable request, subject to patient confidentiality.
